# Supplementary material for: Seafloor video-acoustic monitoring in a Greenlandic glacial fjord records hyperbenthos, backward-swimming fish, and narwhals
Source: PLoS One. 2026 May 6;21(5):e0347193. doi: 10.1371/journal.pone.0347193 (PMC13148681; doi:10.1371/journal.pone.0347193)
Supplement: S1 Fig — The black curve corresponds to the relative area of each image covered by detected particles; the blue curve corresponds to the number of particles, and the red curve corresponds to the mean intensity of the red color. (PDF) [file pone.0347193.s005.pdf]

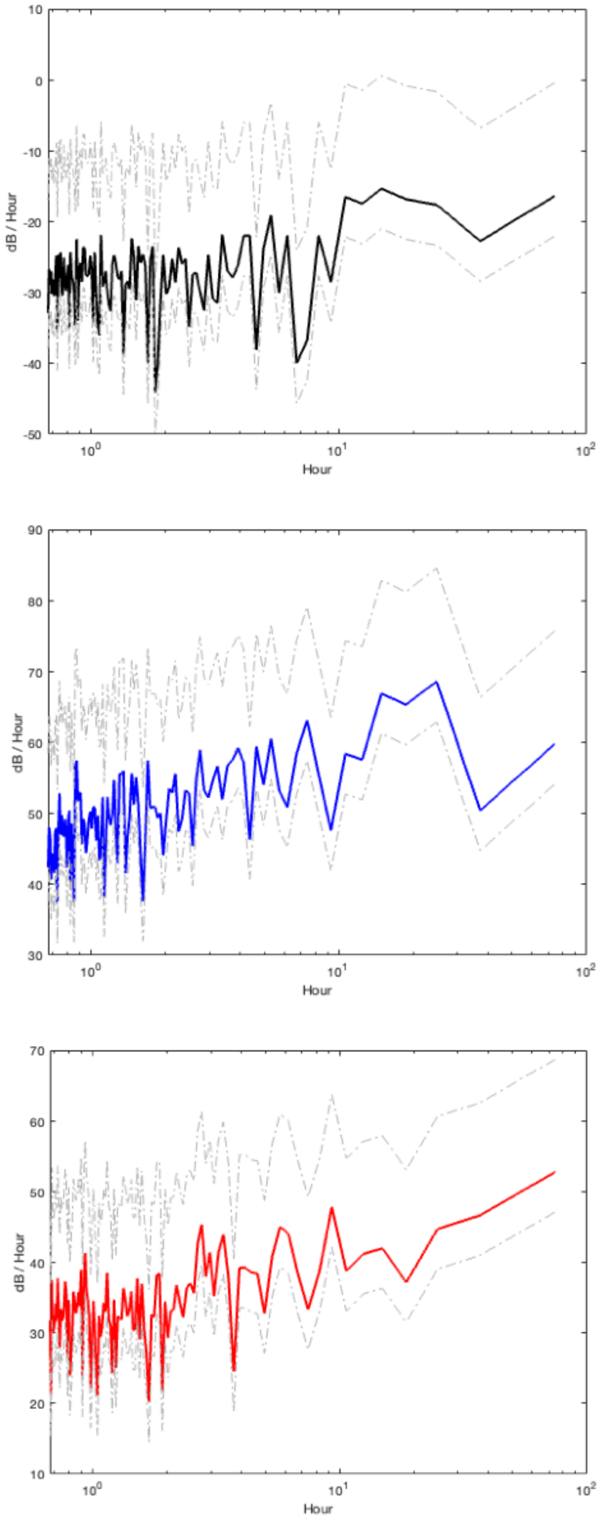

Fig S1. Periodograms with 95%-confidence bounds for extracted image features. The black curve corresponds to the relative area of each image covered by detected particles; the blue curve corresponds to the number of particles, and the red curve corresponds to the mean intensity of the red color.
